# Long non-coding RNA growth arrest specific transcript 5 acts as a tumour suppressor in colorectal cancer by inhibiting interleukin-10 and vascular endothelial growth factor expression

**DOI:** 10.18632/oncotarget.14625

**Published:** 2017-01-13

**Authors:** Yuan Li, Yan Li, Shengkai Huang, Kun He, Mei Zhao, Hong Lin, Dongdong Li, Jiaming Qian, Caihong Zhou, Yuhua Chen, Changzhi Huang

**Affiliations:** ^1^ State Key Laboratory of Molecular Oncology, Cancer Institute and Hospital, Peking Union Medical College and Chinese Academy of Medical Sciences, Beijing, 100021, China; ^2^ Department of Etiology and Carcinogenesis, Cancer Institute and Hospital, Peking Union Medical College and Chinese Academy of Medical Sciences, Beijing, 100021, China; ^3^ Department of Developmental Biology, China Medical University, Shenyang, 110122, China; ^4^ Department of Gastroenterology, Peking Union Medical College Hospital, Chinese Academy of Medical Sciences, Beijing, 100730, China; ^5^ Department of Education, National Cancer Center/Cancer Hospital, Chinese Academy of Medical Sciences and Peking Union Medical College, Beijing, 100021, China

**Keywords:** colorectal cancer, long non-coding RNA, GAS5, interleukin-10, VEGF

## Abstract

Long non-coding RNAs (lncRNAs) are highly involved in diverse biological processes of human malignancies. The expression profile and underlying mechanism of lncRNA growth arrest specific transcript 5 (GAS5) in colorectal cancer (CRC) is poorly understood. In this study, we found that GAS5 was commonly downregulated in CRC tissues, serum of CRC patients and CRC cell lines. Knockdown of GAS5 promoted CRC cell proliferation and colony formation, whereas overexpression of GAS5 produced the opposite result. We further demonstrated that knockdown of GAS5 increased the expression and secretion of interleukin-10 (IL-10) and vascular endothelial growth factor (VEGF-A) via NF-κB and Erk1/2 pathways. Neutralization of IL-10 and VEGF-A reduced tumour proli feration caused by GAS5 knockdown. Moreover, GAS5 expression showed a statistically significant correlation with the mRNA levels of IL-10 and VEGF-A in CRC tissues. We further illustrated that GAS5 was markedly downregulated and negatively correlated with the cytokine expression in a mouse model of colitis-associated cancer (CAC). These results delineate a novel mechanism of lncRNA GAS5 in suppressing colorectal carcinogenesis. The cytokines IL-10 and VEGF-A inhibited by GAS5 may provide targets for lncRNA-based therapies for CRC.

## INTRODUCTION

Colorectal cancer (CRC) is the third most common cancer and the fourth most deadly cancer globally, accounting for approximately 1.2 million new cases and 600,000 deaths annually [1]. Colorectal carcinogenesis is a multistep process. Most cases of CRC are sporadic and develop slowly over 10 years through the adenoma-carcinoma sequence. The 5-year relative survival has reached nearly 65% in high-income countries but has remained under 50% in low-income countries [2]. In the past few decades, numerous molecular pathogenesis studies have indicated that the mutations in certain protein-coding genes (*APC, KRAS, TP53*) correlate with the pathogenesis of CRC [3, 4]. However, recent advances have revealed that long non-coding RNAs (lncRNAs) are also highly involved in the cancer paradigm [5].

LncRNAs, which are mRNA-like transcripts longer than 200 bases that lack protein-coding capability, are reported to be frequently aberrantly expressed in diverse pathological processes in many species [6]. Increasing evidence suggests that lncRNAs serve as key regulators of tumour initiation and development. Some of them have been reported to act as potential diagnostic and prognostic biomarkers for many human malignancies including liver, breast, lung and colon cancers [7–10].

lncRNA GAS5 (growth arrest specific transcript 5) is 651 nucleotides in length and located on chromosome arm 1q25. GAS5 was reported to be upregulated during growth arrest induced by the lack of growth factors or serum starvation [11]. In T-cell lines and human peripheral blood T-cells, overexpression of GAS5 leads to increasing apoptosis and decreasing progression through the cell cycle [12]. Moreover, increasing evidence suggested that GAS5 functions as a tumour suppressor. Several studies have shown that GAS5 is downregulated in tumour tissues compared with corresponding normal tissues, such as breast cancer, renal cell carcinoma, prostate cancer, non-small-cell lung cancer and colorectal cancer [13–17]. However, the biological role and functional mechanism of GAS5 in tumour genesis needs further clarification.

In this study, we primarily focused on CRC and attempted to demonstrate the function of GAS5 in CRC. We not only showed that GAS5 expression was significantly downregulated in CRC tissue relative to adjacent normal tissue but also found that GAS5 expression in the serum of CRC patients was significantly downregulated compared to normal controls. Furthermore, GAS5 overexpression inhibited cell proliferation, colony formation, and cytokine expression and secretion. Our results suggested that decreased GAS5 expression may be important in CRC carcinogenesis.

## RESULTS

### Downregulation of GAS5 in CRC tissues and serum

**Figure 1:**
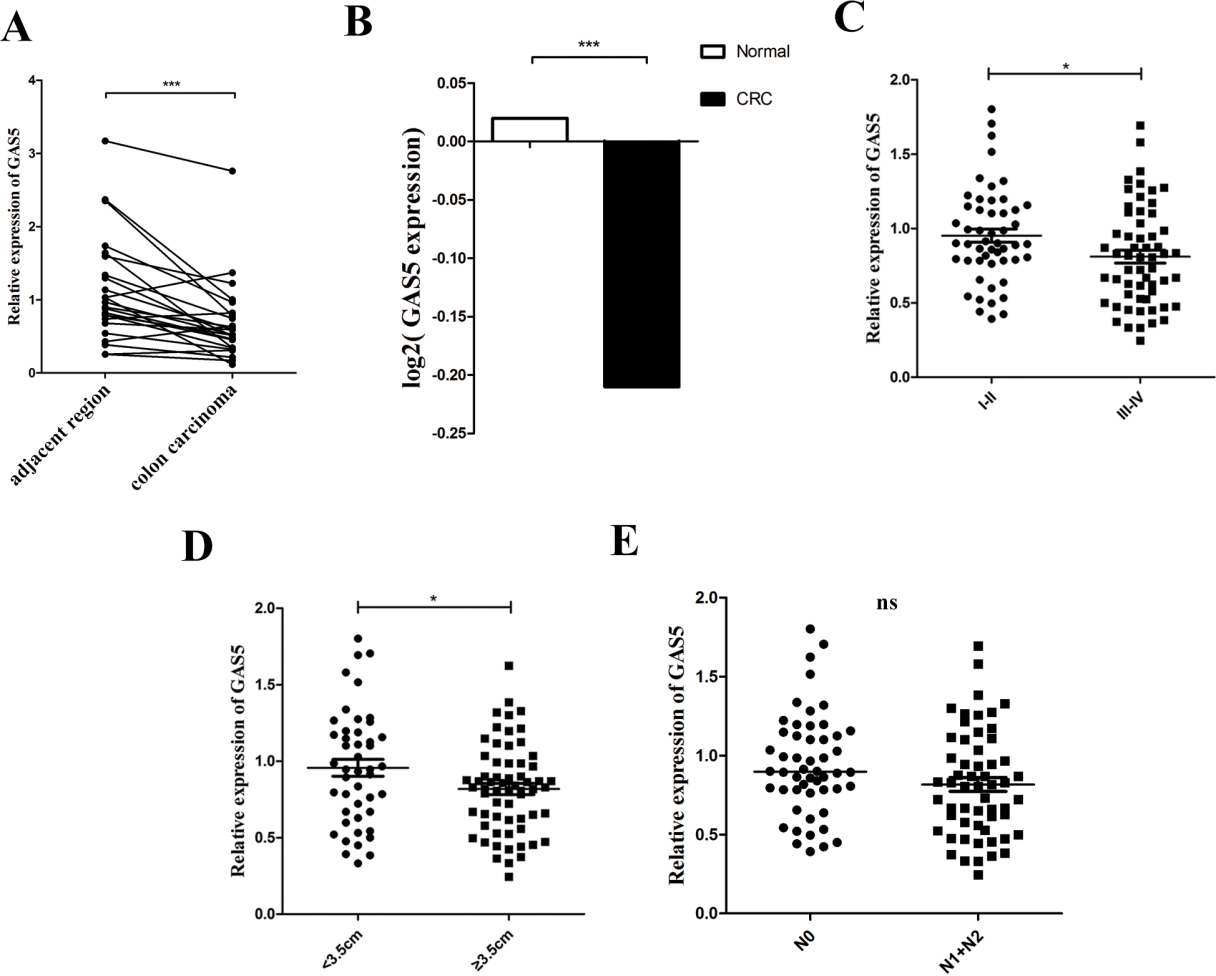
lncRNA GAS5 is downregulated in tissues and serum of patients with CRC (**A**) RT-qPCR analysis of the relative expression of GAS5 in CRC tissues (*n* = 24) and in paired adjacent normal tissues. GAS5 expression was normalized to GAPDH expression. The data are presented as a fold-change in the tumour tissue relative to the normal tissue. ***P < 0.001 by paired t-test (**B**) Circulating RNA was extracted, and GAS5 was measured by RT-qPCR and normalized to GAPDH. Serum GAS5 level showed an obvious clear decrease in patients with CRC (*n* = 109) compared to the normal controls (*n* = 99). The p value was determined by two-sided t test. ***P < 0.001. (**C–E**) Clinical significance of GAS5 in CRC serum. (**C**) GAS5 expression in serum was significantly lower in patients with higher pathological stages. *p* = 0.0268. (**D**) GAS5 expression in serum was significantly lower in patients with tumours ≥ 3.5 cm. *p* = 0.0314. (**E**) GAS5 expression level was not related to lymphatic metastasis. *p* = 0.0508. The p values were determined by two-sided t test. *P < 0.05, **P < 0.01; ***P < 0.001.

The expression of GAS5 was significantly downregulated in the colon carcinomas compared to paired-adjacent normal tissues of 24 patients with sporadic CRC (Figure 1A). An obvious correlation can be seen between GAS5 levels and lymph node invasion, as well as tumour node-metastasis (TNM) staging in patients with CRC; however, no significant correlation between GAS5 levels and sex, age, depth of invasion or tumour size was detected in patients with CRC (Table 1). Furthermore, we examined GAS5 levels in the serum of 109 CRC patients and 99 normal controls. The demographics and clinical characteristics of the study cohort are summarized in Supplementary Table 1. Our result also demonstrated an obvious decrease in GAS5 levels in the serum of CRC patients compared to normal controls (Figure 1B). To further evaluate the correlation between the clinicopathological features and GAS5 expression levels, 109 CRC cases were classified into two groups according to different clinicopathological features. The examination showed that decreased GAS5 expression was tightly correlated with more advanced tumour-node-metastasis (TNM) staging and larger tumour size (Figure 1C–1D) but not lymph node metastasis (Figure 1E). The expression of GAPDH was measured as the endogenous control for serum GAS5 (Supplementary Table 2). The result of the test of lncRNA stability in serum is shown in Supplementary Figure 1A–1C. Collectively, these results suggested that GAS5 might be intimately involved in CRC.

**Table 1:** Association of GAS5 expression with the clinicopathological features of patients with colorectal cancer

Characteristics	GAS5 levels		*p*-value
	low (*n*= 12)	high (*n*= 12)	
Age			
< 55 years	6	4	0.408
≥ 55 years	6	8	
Gender			
Male	7	6	0.682
Female	5	6	
Depth of invasion			
T1–T2	3	2	0.615
T3–T4	9	10	
Tumor size			
≤ 4 cm	3	7	0.141
> 4 cm	9	6	
Lymphatic metastasis			
N0	3	9	0.014*
N1 or above	9	3	
TNM stage			
I–II	3	9	0.014*
xIII–IV	9	3	

Fisher's exact test

### GAS5 inhibits cell proliferation in the CRC cell lines

**Figure 2:**
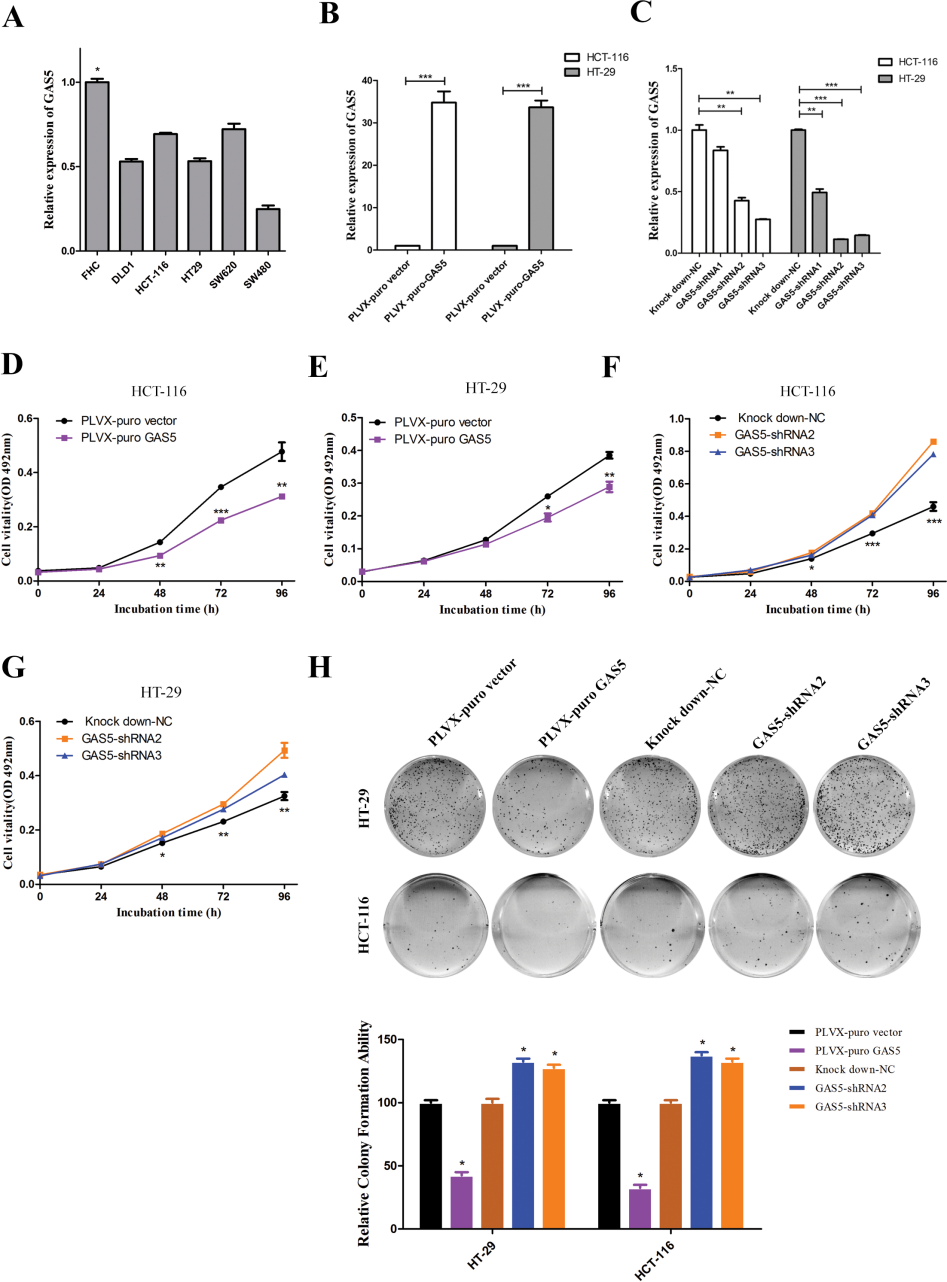
The expression of GAS5 in CRC cell lines and the effect of GAS5 on CRC cell proliferation and colony formation (**A**) GAS5 showed a relatively low expression level in five CRC cell lines (DLD-1, HCT-116, HT-29, SW620, and SW480) compared to FHC, the normal colon tissue cells. The results are shown as the mean ± SEM relative to GAPDH levels from three experiments. *P < 0.05, one-way ANOVA followed by Bonferroni post hoc test. (B–C) Efficacy of stable GAS5 overexpression and knockdown in HCT-116 and HT-29 cells were determined by RT-qPCR. GAPDH was used as an internal control. The results are shown as the mean ± SEM relative to GAPDH levels from three experiments. *P < 0.05, **P < 0.01, ***P < 0.001, compared to the control group by one-way ANOVA followed by Bonferroni post hoc tests. (**D–G**) MTT assays were performed to determine the proliferation of HCT-116 and HT-29 cells after GAS5 manipulation. Stable GAS5 manipulated cells and controls were seeded in 96-well plates and analysed daily for 4 days using the MTT assay. Overexpression of GAS5 (D and E) substantially reduced colorectal cancer cell proliferation, whereas knockdown of GAS5 (**F** and **G**) significantly enhanced the proliferation of both HCT-116 and HT-29 cells. The results are the mean ± SEM from three experiments, and each experiment had six replicates. *P < 0.05; **P < 0.01; ***P < 0.001 compared with control by one-way ANOVA followed by Bonferroni post hoc test. (**H**) Effect of GAS5 overexpression or GAS5 knockdown on the colony formation of HCT-116 and HT-29 cells. The error bar results present the colony formation ability relative to control cells (set to 100%). The values are expressed as the mean ± SEM from three experiments. *P < 0.05 compared to control cells by one-way ANOVA followed by Bonferroni post hoc test.

To understand the role of GAS5 in the progression of CRC, we first used RT-qPCR analysis to assess GAS5 expression in CRC cell lines. As shown in Figure 2A, GAS5 expression was at a low level in five colorectal cancer cell lines, including DLD-1, HCT-116, HT-29, SW620 and SW480, compared with FHC, the normal colon epithelial cells of human. HCT-116 and HT-29 cells were chosen for further mechanistic studies. We transfected HCT-116 and HT-29 cells with lentivirus to generate cell lines that stably expressed the empty vector and full-length GAS5 transcript, as well as cell lines that stably knockdown GAS5 and control cells. RT-qPCR was used to confirm the efficacy of over-expression and knockdown (Figure 2B–2C). Thereafter, we examined the effect of GAS5 manipulation on the proliferation and colony formation of CRC cell lines. Compared to control cells, GAS5 overexpression resulted in a significant decrease in HCT-116 and HT-29 cell proliferation and colony formation abilities (Figure 2D, and 2H). Meanwhile, GAS5 knockdown significantly promoted cell proliferation and colony formation ability in HCT-116 and HT-29 cells (Figure 2F, and 2H).

### GAS5 inhibits expression and secretion of cytokine in HCT-116 and HT-29 cells

The epithelial tumour microenvironment is characterized by a number of inflammatory mediators [18]. Cancer-related inflammation is essential to malignant disease, and inflammatory processes can promote or possibly even initiate malignant disease [19–21]. The autocrine cytokines produced by tumour cells are important mediators that promote tumour progression. Cancer cells of different solid and hematopoietic tumours express multiple growth factors at respective stages of tumour progression, enabling them to grow autonomously through autocrine and paracrine effects [22].

Recently, it was reported that the pro-inflammatory cytokines TNF-α and IL-1α could increase the levels of lncRNA GAS5 in airway epithelial cells [23]. In addition, our data showed that there was clear declining tendency of GAS5 expression in the serum from normal controls to ulcerative colitis patients to CRC patients (Supplementary Figure 1D), suggesting that GAS5 could be related to inflammation. Therefore, we hypothesized that GAS5 may exert its biological effect through regulating specific cytokines.

**Figure 3:**
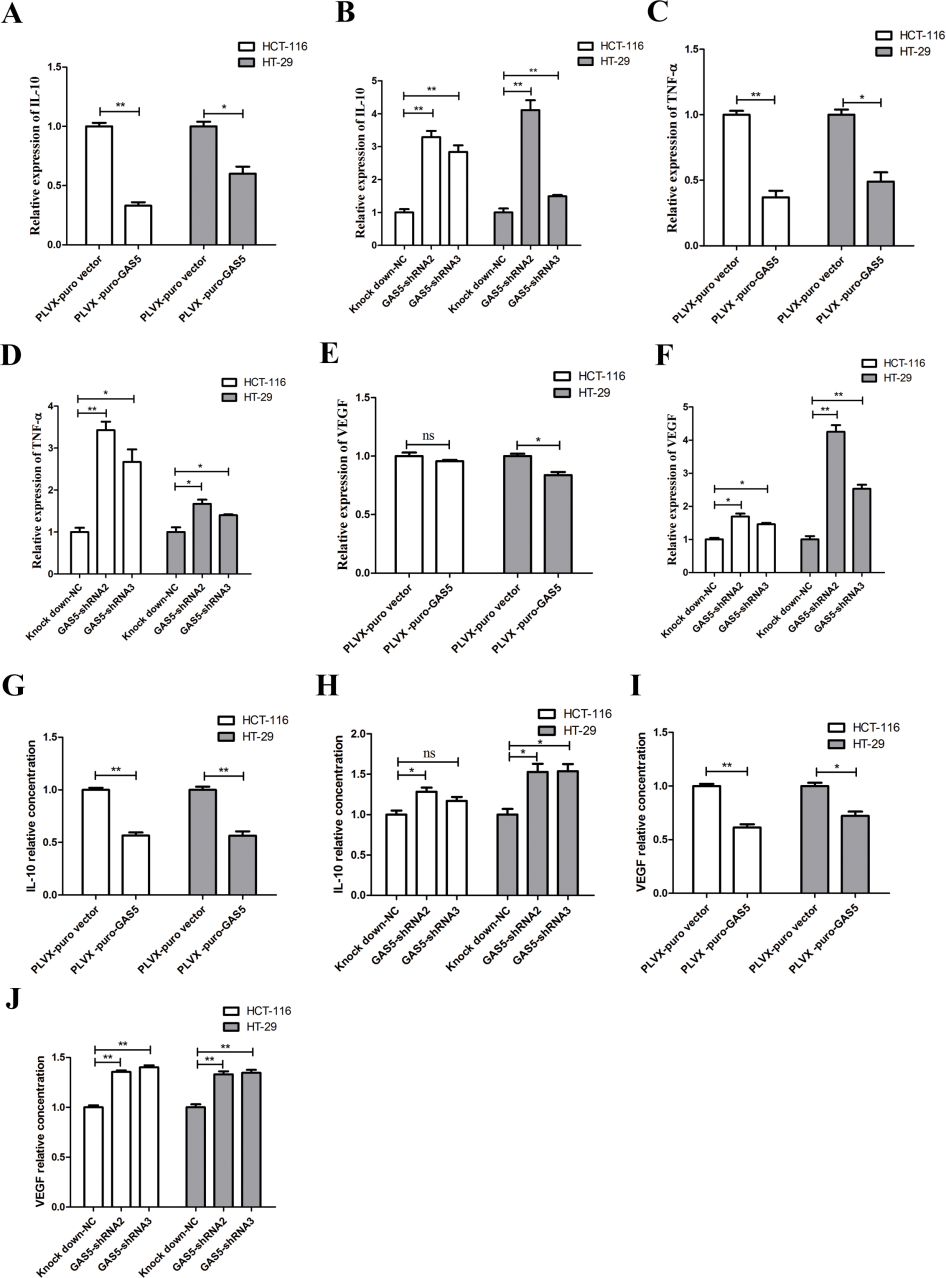
GAS5 inhibits the expression and secretion of cytokines in vitro (**A–F**) RT-qPCR results showed that GAS5 participated in regulating inflammatory gene expression: GAS5 overexpression significantly inhibited IL-10, TNF-α and VEGF-A expression (*P < 0.05; **P < 0.01, two-sided t tests), while GAS5 knockdown significantly promoted IL-10, TNF-α and VEGF-A expression in both HCT-116 and HT-29 cells (*P < 0.05; **P < 0.01, one-way ANOVA followed by Bonferroni post hoc test). The results are shown as the mean ± SEM relative to GAPDH levels from three experiments. (**G–J**) Secretion of inflammatory cytokines was measured by ELISA assay. IL-10 and VEGF-A secretion were clearly decreased in GAS5-overexpressing cell lines (*P < 0.05; **P < 0.01, two-sided t tests). IL-10 and VEGF-A secretion was highly increased in GAS5 knockdown cell lines (*P < 0.05; **P < 0.01, one-way ANOVA followed by Bonferroni post hoc test). The results are shown as the mean ± SEM relative to the control group from three experiments.

Using stable GAS5 over-expression and knockdown cell lines, we performed RT-qPCR for several types of cytokines, including TNF-α, TGF-β, IL-1α, IL-6, IL-10, IL-11, IL-17A, IFNA1 and VEGF-A, all of which are highly involved in CRC tumourigenesis. RT-qPCR results revealed a significant increase in interleukin-10 (IL-10), tumour necrosis factor alpha (TNF-α) and vascular endothelial growth factor (VEGF-A) in HCT-116 and HT-29 GAS5 knockdown cells compared with control cells. Meanwhile, HCT-116 and HT-29 GAS5 overexpression cells produced significantly less IL-10, TNF-α and VEGF-A than their counterparts (Figure 3A–3F). We further validated the result by ELISA assay using cell culture supernatant. VEGF-A and IL-10 secretion amount showed consistency with the RT-qPCR results (Figure 3G–3J), while TNF-α secretion levels in the supernatant of HCT-116 and HT-29 cells were somehow lower than the Lower Limit of Detection of the ELISA kit.

From the results above, we drew the conclusion that GAS5 can inhibit specific cytokine expression and secretion *in vitro*.

### GAS5 inhibits cytokine secretion associated with NF-κB and MAPK/ERK pathway

**Figure 4:**
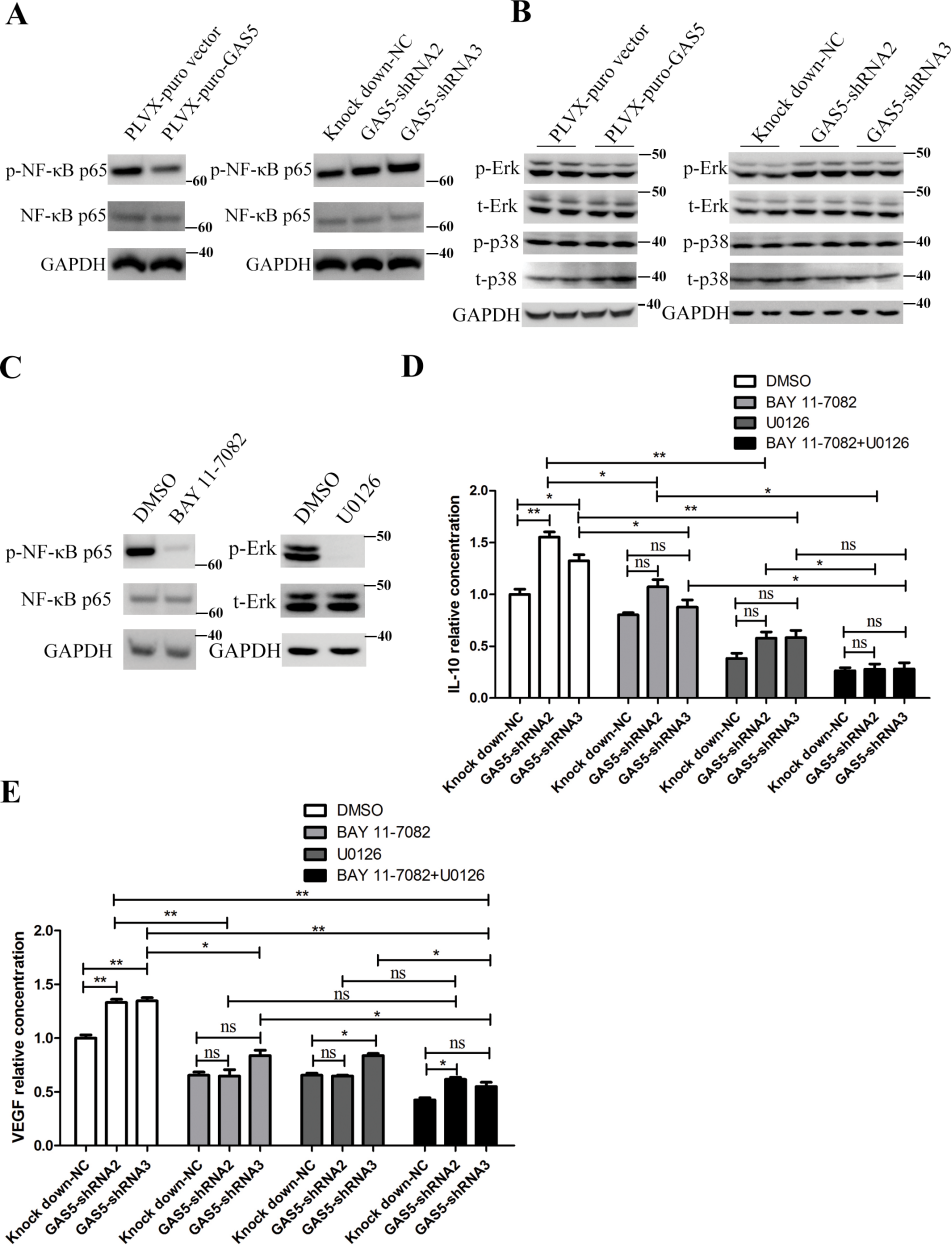
GAS5 inhibits the secretion of inflammatory cytokines via the NF-κB and Erk1/2 MAPK pathways (**A**) GAS5 overexpression inhibited p-NF-κB p65, and GAS5 knock-down and significantly enhanced p-NF-κB p65 in HCT-116 cells. (**B**) GAS5 overexpression inhibited phosphorylated Erk1/2, and GAS5 knockdown significantly enhanced phosphorylated Erk1/2 in HCT-116 cells. p38 MAPK remained stable. (**C**) HCT-116 cells were treated with 10 μM U0126 or 10 μM BAY 11-7082 or DMSO, and the levels of p-Erk, total Erk1/2 (t-Erk), p-NF-κB p65 and total NF-κB p65 were measured. GAPDH was used as a loading control. (**D–E**) ELISA assays were performed to evaluate the secretion of IL-10 and VEGF-A in the supernatant of HCT-116 cells after different treatments. The results are shown as the mean ± SEM relative to the NC group from three experiments. *P < 0.05; **P < 0.01, compared to the control group, and all P values are from one-way ANOVA followed by Bonferroni post hoc tests.

As the expression of several inflammation-related genes was inhibited, we next asked what are the intracellular mechanisms that mediate the effects of GAS5? NF-κB is a critical link between inflammation and cancer. Aberrant NF-κB activation can promote cancer invasion and metastasis. Therefore, we tested whether over-expression and knockdown of GAS5 affected the level of phosphorylated NF-κB p65 (p-NF-κB p65). As shown in Figure 4A, GAS5 overexpression significantly decreased the level of p-NF-κB p65, and GAS5 knockdown obviously increased the level of p-NF-κB p65 without affecting the level of total NF-κB p65 in HCT-116 cells.

The p38 mitogen-activated protein kinase (MAPK) plays important roles in the cellular response to stress stimuli, including cytokines and heat shock, and is involved in cell apoptosis, differentiation and cytokine production [24]. We explored the possibility that GAS5 function through p38 in HCT-116 cells. However, phosphorylation of p38 was not affected in GAS5-overexpressing and knockdown cells (Figure 4B). Additionally, extracellular signal-regulated kinase 1/2 is another major subfamily of MAPKs in transducing intracellular signalling [24]. We continued to investigate whether GAS5 function through Erk1/2. We measured the levels of phosphorylated and total Erk1/2 in GAS5-overexpressing and knockdown cells. The results showed that GAS5 overexpression significantly inhibited Erk1/2 phosphorylation and that GAS5 knockdown clearly increased Erk1/2 phosphorylation without affecting the level of total Erk1/2 (Figure 4B).

Consequently, we focused on investigating whether NF-κB and MAPK/ERK signalling are responsible for dysregulated cytokine secretion in stable GAS5 over-expression and knockdown cells. We used BAY 11-7082, an inhibitor of NF-κB nuclear translocation, and U0126 (MEK1/2 inhibitor), a selective MAPK kinase inhibitor, to inhibit NF-κB and Erk1/2 signalling activation, respectively. ELISA results showed that both BAY 11-7082 and U0126 significantly abrogated GAS5 knockdown-induced VEGF-A and IL-10 secretion upregulation in HCT-116 cell. Additionally, we used BAY 11-7082 together with U0126, and the result showed that the level of cytokine secretion continued to decline compared to BAY 11-7082 inhibition alone or U0126 inhibition alone (Figure 4C–4E).

These results together demonstrated that NF-κB and MAPK/ERK signalling both participate in the regulation of cytokine expression and secretion by GAS5.

### Neutralization of cytokines reduces tumour proliferation caused by GAS5 knockdown

**Figure 5:**
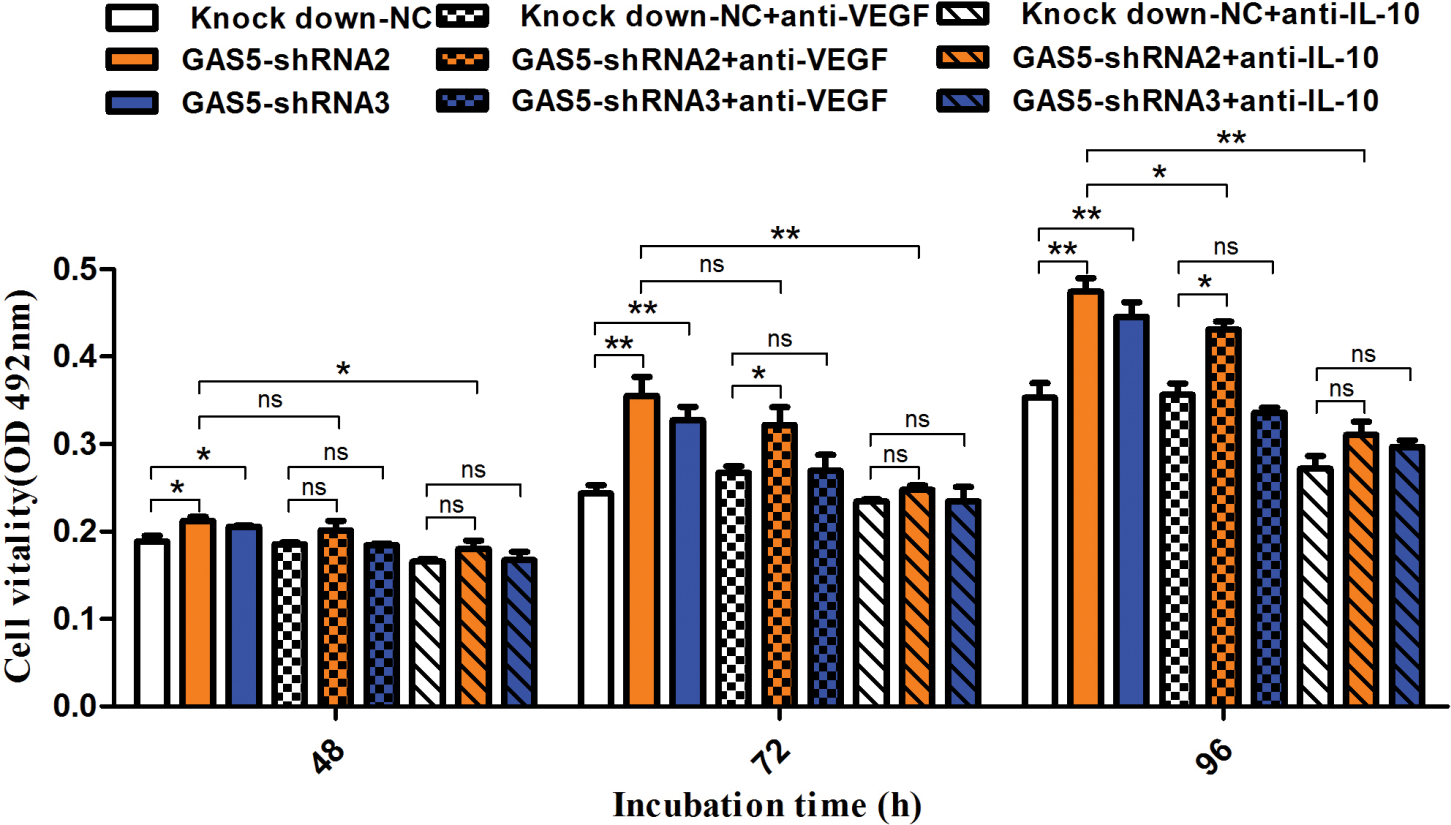
Neutralization of IL-10 and VEGF-A reduces tumour proliferation caused by GAS5 knockdown Neutralization antibody against IL-10 (1.5 μg/ml) or VEGF-A (0.1 μg/ml) was incubated with tumour cells, and MTT assays were performed at different time points (48 h, 72 h, and 96 h) to test tumour cell proliferation. The results are shown as the mean ± SEM from three experiments. *P < 0.05; **P < 0.01, compared to the control group, and all P values are from one-way ANOVA followed by Bonferroni post hoc tests.

From the results above, GAS5 can inhibit both cell proliferation and cytokine secretion. To investigate the contribution of specific cytokines to the proliferation of tumour cells, we performed MTT assays in the presence of neutralizing antibodies to both VEGF-A and IL-10. MTT assays were performed at different time points to test tumour cell proliferation, and the results showed that neutralization of IL-10 (1.5 μg/ml) attenuated tumour cell proliferation significantly at 48 h, 72 h and 96 h. Importantly, compared to the control group, neutralization of IL-10 eliminated the abnormally increased proliferation caused by GAS5 knockdown (Figure 5). However, neutralization of VEGF-A (0.1 μg/ml) induced a relatively mild downregulation of cell proliferation at these time points and showed a partial rescue effect.

### GAS5 inhibits cytokine expression in human CRC and murine CAC

**Figure 6:**
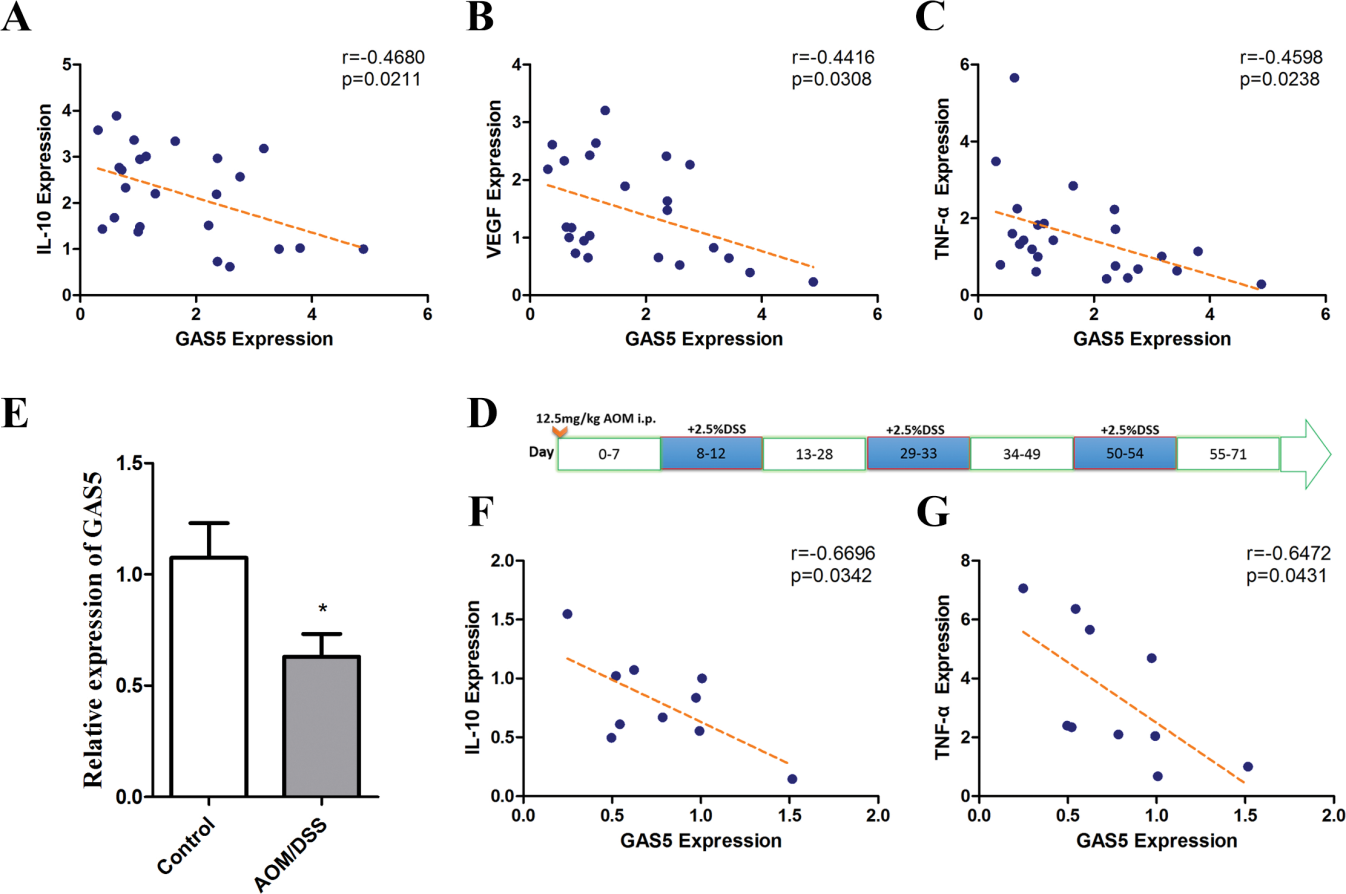
Correlations of GAS5 levels with IL-10, VEGF-A and TNF-α in colorectal tumour tissues and GAS5 downregulation in the CAC mouse model (**A–C**) The RNA levels were determined by RT-qPCR relative to GAPDH. A significant negative correlation was observed between GAS5 expression and mRNA levels of IL-10, VEGF-A and TNF-α in CRC samples (n = 24). The r values and P values are from Pearson's correlation analysis. (**D**) Diagram of AOM/DSS administration schedule for establishing the colitis-associated colon cancer model. (**E**) GAS5 downregulation in the colon of wild type (WT) mice on day 71 of AOM/DSS administration compared with the control group (*n* = 10). The results are shown as the mean ± SEM relative to GAPDH levels from three experiments. *P < 0.05, compared to the control group by two-sided t tests. (**F–G**) Negative correlations between GAS5 and IL-10 or TNF-α were detected in the colon of the CAC mouse model (*n* = 10). The inset shows the Pearson's r correlation and corresponding *P* value.

Because we found that GAS5 could inhibit IL-10, VEGF-A and TNF-α expression *in vitro*, we analysed this effect *in vivo*. GAS5 expression showed a significant negative correlation with the mRNA levels of IL-10, VEGF-A and TNF-α in CRC samples (Figure 6A–6C).

We next detected the levels of GAS5 in the AOM/DSS-induced CAC (colitis-associated cancer) mouse model, which is induced by the repeated sequential administration of azoxymethane (AOM) and dextran sulfate sodium (DSS). In this CAC model, it has been reported that neutralization of the cytokine TNF-α [25] and the transcription factor NF-κB [21] reduces tumour proliferation. Thus, we expected that GAS5 may also be downregulated and related to the expression of cytokines in the CAC model.

Consequently, WT mice were treated with DSS/AOM (Figure 6D) and sacrificed on day 71 (*n* = 10), the colons were resected, and the expression level of GAS5 was detected. After AOM/DSS administration, GAS5 expression in the colon was significantly decreased compared with that of the control group (Figure 6E). Moreover, the expression of GAS5 was negatively correlated with IL-10 and TNF-α (Figure 6F–6G).

In summary, these results demonstrated that GAS5 is negatively correlated with the expression of specific cytokines *in vitro* and *in vivo*.

## DISCUSSION

Here, we showed that GAS5 is downregulated not only in tumour tissues but also in the serum of patients with CRC. In addition, we demonstrated that GAS5 downregulation correlated with larger tumour size and more advanced tumour-node-metastasis (TNM) staging in the serum of CRC patients. Our result suggested that GAS5 may be highly involved in the progression of CRC.

The stimulating effect of inflammation on cancer is better understood and more widely accepted [26, 27]. Immune cells densely infiltrate tumours and preneoplastic lesions, release bioactive molecules to the tumour microenvironment, including various cytokines and chemokines that propagate localised inflammatory conditions, and promote the growth and survival of premalignant cells. More importantly, in some cases, inflammation is evident at the earliest stages of neoplastic progression. Furthermore, inflammation is demonstrably able to foster the development of incipient neoplasias into full-blown cancers [28, 29].

Tumour cells can also secrete cytokines. In this study, in HCT-116 and HT-29 cells, we found that GAS5 significantly inhibit the release of IL-10 and VEGF-A, which indicates that GAS5 is involved in the tumour-associated inflammatory response.

Many different types of cells can produce IL-10, including T lymphocytes, activated macrophages, B cells, dendritic cells, mast cells, and intestinal epithelial cells [30–32]. Moreover, IL-10 is elevated in cancer and is thought to contribute to tumour growth and immune tolerance [33]. Sredni et al. reported that IL-10 is essential for tumour cell proliferation in a murine B16 melanoma cell line and two human primary cultures of stomach adenocarcinoma and glioblastoma (GBM), as its neutralization decreases the clonogenicity of malignant cells, whereas the addition of recombinant IL-10 increases cell proliferation [22]. In human renal cell carcinoma specimens, IL-10 is associated with a poor prognosis, and patients with elevated IL-10 serum levels have a worse outcome after surgery [34]. Autocrine secretion of IL-10 neutralizes CD95-generated signals and allows the survival and growth of thyroid cancer cells [35]. Furthermore, autocrine secretion of IL-10 promotes thyroid tumour cell progression and resistance to chemotherapy through the up-regulation of anti-apoptotic proteins [36].

Vascular endothelial growth factor (VEGF-A) is a well-known angiogenesis inducer [37]. Tumour cells secrete VEGF-A primarily through autocrine and paracrine mechanisms. Previous studies have confirmed that VEGF-A produced by malignant tumour cells binds to VEGF receptors (VEGFR)2 on the adjacent vascular endothelial cells, promoting vascular endothelial cell division and proliferation and inducing tumour angiogenesis, growth and metastasis [38, 39]. In recent years, studies have shown that some malignant tumour cells can selectively express functional VEGFR, whereby VEGF-A produced by tumour cells can bind to receptors of their own, promoting tumour cell proliferation and invasion [40, 41]. Consistent with these previous reports, our study showed that GAS5 suppresses CRC progression through regulating IL-10 and VEGF-A.

Signalling pathways are critical for the secretion of inflammatory cytokines in tumour cells. In our study, we identified the NF-κB and Erk1/2 pathways as the underlying mechanisms through which GAS5 inhibits the inflammatory profile in colorectal cell lines. A previous study suggested that Erk1/2 may be involved in the activation of NF-κB through promoting IκB kinase activation and IκB degradation in LPS-stimulated macrophages [42]. Thus, it is possible that downregulation of GAS5 additionally activates an Erk1/2/NF-κB pathway to enhance the inflammatory effects in tumour cells.

Using the lncRNA array carrying human disease-related lncRNAs, Zhang et al. showed that miR-21 is capable of suppressing the lncRNA GAS5 through a putative miR-21-binding site in exon 4 of GAS5 and that GAS5 can also repress miR-21 expression. This negative correlation has also been seen in breast tumour specimens [43]. Moreover, miR-21 levels were markedly upregulated in the tumours of patients with CRC or CAC and in a mouse model of CAC [44, 45]. Following AOM and DSS intervention, miR-21-knockout mice showed a decrease in the size and number of tumours compared with the control group. The absence of miR-21 also reduced the expression of inflammatory cytokines (IL-6, IL-23, IL-17A and IL-21) and attenuated the proliferation of tumour cells [44]. All of these reports support our results indirectly.

In summary, this study reveals the crucial involvement of GAS5 in CRC and demonstrates that GAS5 acts as an important regulator of the genesis and development of CRC through inflammatory cytokines via NF-κB and Erk1/2 pathways.

## MATERIALS AND METHODS

### Ethics statement and patients

Written informed consent was obtained from each patient and healthy volunteer before the study, and all of the tumour patients’ and healthy volunteers’ samples were collected at the Cancer Hospital, Chinese Academy of Medical Sciences between 2013 and 2014. All of the CRC patients in the study had pathologically and histologically confirmed diagnoses. The tumours were staged according to the tumour-node-metastasis (TNM) staging system of the Seventh Edition of the Union for International Cancer Control. Healthy controls were free of known malignancy or active inflammatory condition and were age- and gender-matched to the patients.

Human colon cancer tissues and paired normal tissues (5 cm adjacent to tumour) were obtained from 24 patients. Cancer tissues and matched normal tissues were dissected and submerged in RNAlaterTM (Ambion) for 24 h and then stored at –80°C until use. All the patients received no chemotherapy before surgical resection for colon cancer and signed informed consent forms for sample collection.

### Serum collection and RNA isolation

Peripheral blood samples (< 5 ml), obtained from another 109 CRC patients, were collected into serum collection tubes, and allowed to clot at room temperature for approximately 1 h before a two-step centrifugation (820 g for 10 min at 4°C, followed by 16,000 g for 10 min at 4°C) to completely remove any cell debris. Serum samples were transferred to RNase-free DNase-free tubes and stored at −80°C until total RNA extraction.

Total RNA in serum was isolated using Trizol LS Reagent (Invitrogen, Carlsbad, CA, USA). For each 250 μl of serum, 750 μl of Trizol LS was added for phase separation, followed by 200 μl of chloroform to augment the RNA phase separation process. Total RNA was precipitated by isopropanol, washed with 75% ethanol and solubilized in 30 μl of RNase-free water.

RNA extraction from cultured cells was performed using Trizol reagent (Invitrogen) according to the manufacturer's instructions.

### Reverse transcription and quantitative PCR (RT-qPCR)

RNA reverse transcription was performed using the TaKaRa reverse transcriptase M-MLV kit (2641A; Takara) according to the manufacturer's protocol. RT-qPCR was carried out using SYBR Premix Ex Tag™ II (RR820A; Takara) in 20-μl reaction volumes. RT-qPCR reactions were performed on an ABI 7500 Real-Time PCR System (Applied Biosystems, USA). Each experiment was repeated three times, and the specificity of each PCR reaction was confirmed by melt curve analyses.

The expression of GAPDH was measured as an endogenous control for serum (Supplementary Table 2) and cellular lncRNAs. All of the samples were normalized to the endogenous control according to the 2^−ΔΔCT^ method. All of the primers used in the present study are listed in the supporting information (Supplementary Table 3).

### Induction of colitis-associated cancer

Colitis-associated cancer (CAC) modelling was performed as previously described [46]. Briefly, six- to eight-week-old male mice were injected i.p. with AOM (12.5 mg/kg; Sigma-Aldrich). On day eight after AOM intervention, the mice were treated with 2.5% DSS (MW 36,000–50,000 Da; MP Biomedicals) in drinking water for five consecutive days, followed by regular drinking water for 16 days. DSS treatment was repeated for two additional cycles. During the course of treatment, the mice were weighed and monitored for diarrhoea and haematochezia. On day 71, colon tissues were opened longitudinally and measured. Macroscopically visible tumours were quantified. Subsequently, colon sections were fixed in 10% formalin and paraffin embedded.

### Cell culture

HCT-116 and HT-29 cell lines were purchased from ATCC. The HCT-116 cells were cultured in DMEM medium and the HT-29 cells in DMEM/F12 medium. Both cell lines were maintained in basic medium supplemented with 10% foetal bovine serum and penicillin/streptomycin in a humidified atmosphere of 5% CO_2_ at 37°C.

### Generation of GAS5 knockdown and overexpression and control cell lines

The GAS5-specific short hairpin RNA (shRNA) templates were cloned into the pSIH-H1 shRNA Cloning and Expression Vector. The interference sequences targeting GAS5 were as follows: 5′-shRNA1-CTTGCCT GGACCAGCTTAATT-3′, 5′-shRNA2-TATGGAGAGT CGGCTTGACTACACTGTGT-3′ and 5′-shRNA3-TT GGCACACAGGCATTAGACAGAAAGCTG-3′. The scramble sequence 5′-GTTCTCCGAACGTGTCACGT-3′ was used as the negative control (NC). Lentivirus particles were prepared by co-transfecting specific pSIH-H1 shRNA Vector with packaging plasmids (pMD2.G and psPAX2) into 293T cells using PEI transfection reagent. Virus was harvested 48 h and 72 h after transfection. After transfection with lentivirus particles, HCT-116 and HT-29 cells stably expressing the specific short hairpin RNA and control were selected in media containing 2 μg/ml puromycin.

The GAS5 sequence was synthesized according to the full-length GAS5 sequence (based on the GAS5 sequence, NR_002578, in NCBI) and cloned into a pLVX-puro lentiviral expression vector. The empty pLVX-puro lentiviral expression vector was used as a control. Similarly, HCT-116 and HT-29 cells stably expressing GAS5 and control were selected with puromycin after lentivirus transfection.

### MTT assay

Cells were seeded in 96-well plates with 2000 cells in 100 μl per well. Cells were maintained and determined daily for 4 days. Cell viability was determined every 24 h. Briefly, 20 μl of MTT solution (5 mg/ml, Sigma, USA) combined with 90 μl DMEM was added to cells, and then incubate at 37°C for 3 h. Absorbance at OD 492 nm was determined with a microplate reader. Each experiment with six replicates was repeated three times.

### Colony formation

For colony formation assays, cells were seeded into six-well plates with 1000 cells per well and maintained in complete medium containing 10% FBS. Cells were allowed to grow until visible colonies formed (2 weeks). Then, cells colonies were fixed with methanol, stained with Giemsa solution and counted. Each well was assessed in triplicate.

### Determination of cytokine production by ELISA

Tumour cells (1 × 10^4^) were seeded in a 96-well plate, and the supernatants were collected after 24 h. The production of cytokines in the supernatant was measured by ELISA according to the manufacturer's guideline (CUSABIO BIOTECH CO., Ltd., Wuhan, China).

### Western blot

Cells were harvested in lysis buffer [50 mM Tris-HCl, pH 7.4, 1.5% NP-40, 0.1% SDS, 150 mM NaCl, 50 μg/ml PMSF, with fresh proteinase inhibitor cocktail (Roche)]. Proteins from total cell lysates were separated by SDS-PAGE, transferred to PVDF membranes, blocked in 5% bovine serum albumin (BSA) in TBST, and blotted with primary antibody according to the manufacturer's instructions [GAPDH, 1:5000, Sigma; Erk, 1:1000, Cell Signaling Technology; Phosphor-Erk, 1:1000, Cell Signaling Technology; P38, 1:1000, Cell Signaling Technology; Phosphor-P38, 1:1000, Cell Signaling Technology; NF-κB p65, 1:1000, Cell Signaling Technology; Phosphor-NF-κB p65(Ser536), 1:1000, Cell Signaling Technology]. Thereafter, the membranes were washed three times with TBST and incubated with appropriate secondary antibodies for 1 h at room temperature. The ECL chemiluminescence system was used to detect the signals.

### Statistical analyses

The Fisher's exact test was used to analyse the relationship between GAS5 levels in tumour tissues and the clinicopathological parameters. The Paired *t* test was used to compare the GAS5 expression between paired colon carcinoma and adjacent region. The differences in GAS5 expression in serum between patients and healthy individuals were evaluated by the Student's *t* test. For the functional analyses, results are presented as means ± SEM; the comparison of means between two groups was conducted using Student's *t* test, and comparison for more than two groups was conducted using one-way ANOVA followed by Bonferroni post hoc test. Multiple correlation analysis was assessed by Pearson's test. A *p value* less than 0.05 was considered as statistical significance. SPSS (version 13.0) and GraphPad Prism 5.0 were used for data analysis.

## SUPPLEMENTARY MATERIALS FIGURES AND TABLES


