# Supplementary material for: Long non-coding RNA growth arrest specific transcript 5 acts as a tumour suppressor in colorectal cancer by inhibiting interleukin-10 and vascular endothelial growth factor expression
Source: Oncotarget. 2017 Jan 13;8(8):13690–702. doi: 10.18632/oncotarget.14625 (PMC5355130; doi:10.18632/oncotarget.14625)
Supplement: Supplementary file 1 [file oncotarget-08-13690-s001.pdf]

Long non-coding RNA growth arrest specific transcript 5 acts as a tumour suppressor in colorectal cancer by inhibiting interleukin-10 and vascular endothelial growth factor expression

Supplementary Materials

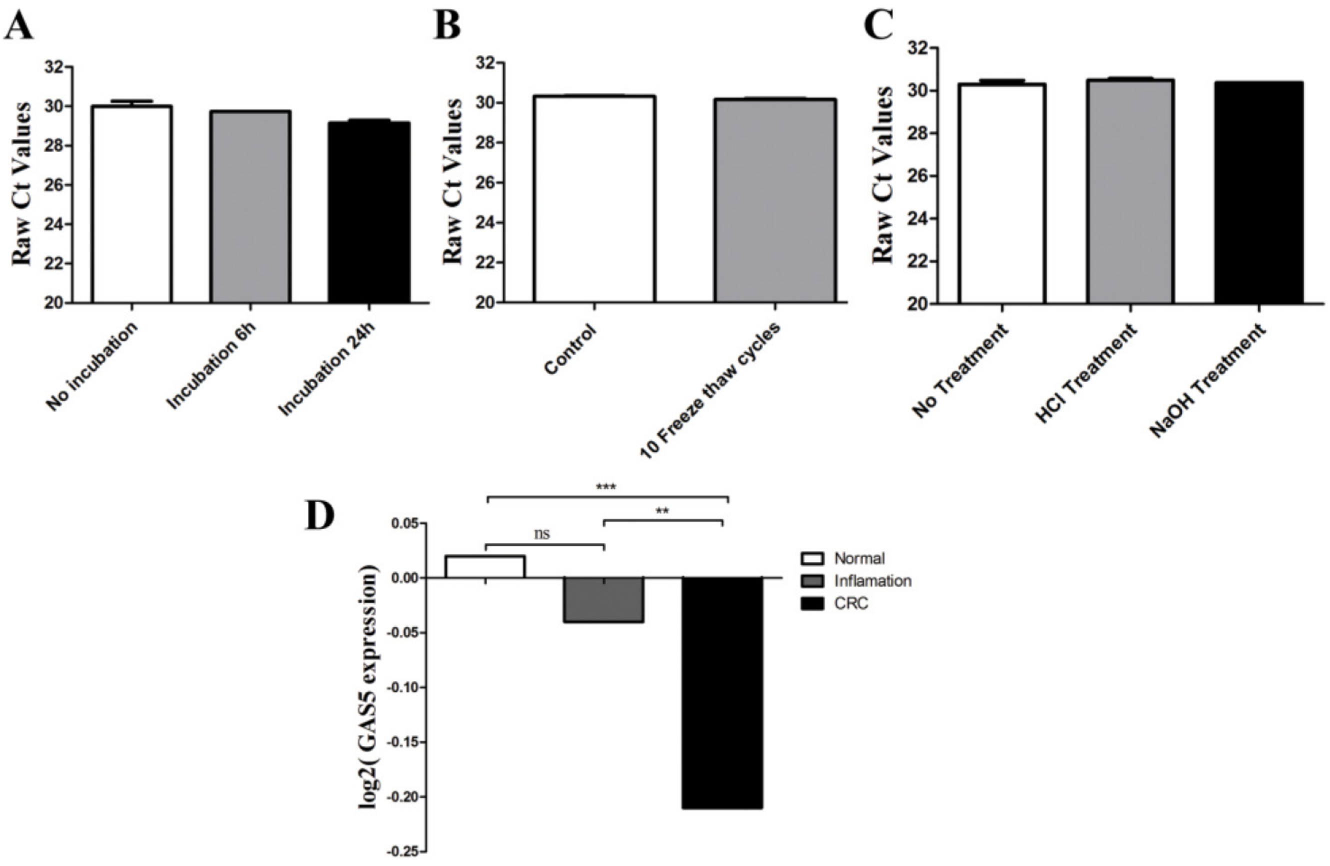

Supplementary Figure 1: Stability of serum lncRNA GAS5 and GAS5 expression profile in the serum of normal people, ulcerative colitis patients and CRC patients. GAS5 showed no significant degradation when serum was treated with (**A**) prolonged room temperature incubation time, or (**B**) multiple freeze-thaw cycles, or (**C**) low (pH = 1) or high (pH = 13) pH solution. Data was presented as raw Ct value, and each bar represented the mean (SD) (n = 3). (**D**) Circulating RNA was extracted and GAS5 was measured by RT-qPCR, then normalized to GAPDH. There was an obvious decline tendency of GAS5 expression in serum from normal controls (n = 99) to ulcerative colitis patients (n = 74), then to CRC patients (n = 109).

Supplementary Table 1: Clinical characteristics of 99 healthy controls and 109 CRC patients

Characteristics		Healthy controls $n = 99$	CRC cases $n = 109$	p-value
**Age**	< 55 years	30	36	0.673
	≥ 55 years	69	73	
**Gender**	Male	54	63	0.637
	Female	45	46	
**Smoking status**	Ever or current	24	29	0.696
	Never	75	80	
**Alcohol consumption**	Ever or current	27	31	0.851
	Never	72	78	
**TNM stage**	I	-	14	
	II	-	37	
	III	-	43	
	IV	-	15	
**Family history of tumor**	Yes	-	10	
	No	-	99	
**Carcinoembryonic antigen (CEA)**	Positive	-	25	
	Negative	-	84	
**Carbohydrate antigen19-9(CA19-9)**	Positive	-	10	
	Negative	-	99	
**Tumor size**	< 3.5 cm	-	48	
	≥ 3.5 cm	-	61	
**Lymphatic metastasis**	Absent	-	51	
	Present	-	58	

Chi-squared test.

Supplementary Table 2: Correlation between GAPDH level (raw Ct value) in human serum and clinicopathological factors of healthy controls and CRC patients

Variable	No. of patients	*GAPDH* level (Mean ± SD)	p-value
Age (years)			
< 55	66	32.72 ± 1.318	0.705
≥ 55	142	32.79 ± 1.203	
Gender			
Male	117	32.68 ± 1.244	0.231
Female	91	32.88 ± 1.227	
Pathological diagnosis			
CRC	109	32.91 ± 1.375	0.075
Healthy controls	99	32.61 ± 1.051	

Supplementary Table 3: Primers used in the current study

GAPDH	forward 5′-3′	ACAACTTTGGTATCGTGGAAGG
	reverse 5′-3′	GCCATCACGCCACAGTTTC
GAS5	forward 5′-3′	AAGCCATTGGCACACAGGCATTAG
	reverse 5′-3′	AGAACCATTAAGCTGGTCCAGGCA
TNF-α	forward 5′-3′	GAGGCCAAGCCCTGGTATG
	reverse 5′-3′	CGGGCCGATTGATCTCAGC
IFNA1	forward 5′-3′	GCCTCGCCCTTTGCTTTACT
	reverse 5′-3′	CTGTGGGTCTCAGGGAGATCA
IL6	forward 5′-3′	CCTGAACCTTCCAAAGATGGC
	reverse 5′-3′	TTCACCAGGCAAGTCTCCTCA
IL10	forward 5′-3′	GACTTTAAGGGTTACCTGGGTTG
	reverse 5′-3′	TCACATGCGCCTTGATGTCTG
TGFB1	forward 5′-3′	GGCCAGATCCTGTCCAAGC
	reverse 5′-3′	GTGGGTTTCCACCATTAGCAC
VEGFA	forward 5′-3′	AGGGCAGAATCATCACGAAGT
	reverse 5′-3′	AGGGTCTCGATTGGATGGCA
IL1A	forward 5′-3′	AGATGCCTGAGATACCCAAAACC
	reverse 5′-3′	CCAAGCACACCCAGTAGTCT
IL17A	forward 5′-3′	TCCCACGAAATCCAGGATGC
	reverse 5′-3′	GGATGTTCAGGTTGACCATCAC
IL11	forward 5′-3′	CGAGCGGACCTACTGTCCTA
	reverse 5′-3′	GCCCAGTCAAGTGTCAGGTG
